# Temporal trend, clinicopathologic and sociodemographic characterization of age at diagnosis of breast cancer among US women diagnosed from 1990 to 2009

**DOI:** 10.1186/2193-1801-3-626

**Published:** 2014-10-23

**Authors:** Gabriel Escarela, Luis Carlos Pérez-Ruiz, Gabriel Núñez-Antonio

**Affiliations:** Departement of Mathematics, Universidad Autónoma Metropolitana - Iztapalapa, AT-351 UAM-I Av. San Rafael Atlixco No. 186 Col. Vicentina, Mexico City, DF 09340 Mexico

**Keywords:** Bimodality, Breast cancer etiology, Clustering, Histopathology, SEER, Split-population model

## Abstract

This paper investigates the distribution of age at diagnosis of female breast cancer and its association with temporal trend, clinicopathologic and sociodemographic variables in the presence of two latent clusters that are directly unobservable. Such clusters help to identify two subpopulations of either young or old patients whose etiologies are thought to be different. A large sample drawn from registry data from the National Cancer Institute’s Surveillance, Epidemiology, and End Results program from 1990 to 2009 was analyzed using a two-component Gaussian mixture model. Evidence of a steady delay of age at diagnosis and an increasing proportion of young patients being diagnosed during the 20-year period was found. Histopathologic effects indicate that duct and lobular carcinomas differ significantly in regard to subpopulation membership, which confirms that they represent different etiologies. While the presence of estrogen receptor status in the model overlaps the effects of other important variables it is highly correlated with, it is found that the grade, extension and size of the tumor along with lymph node involvement status, race and marital status are important predictors of age at diagnosis. The results highlight the significant impacts that such features can have on breast cancer control efforts, and point to the importance of ensuring that medical decision making should use them along with an indicator of the age subpopulation a patient may belong to.

## 1 Introduction

Female breast cancer is a complex disease with different clinicopathologic features. Over the past three decades in the US, diagnostic methods have changed and female breast cancer etiology has evolved due to change in lifestyles and frequent screening. One of the primary challenges of breast cancer epidemiology is to identify risk factors such as age at onset which may provide clues for understanding the etiologic mechanisms of the disease. The ability to discriminate etiologically different subgroups in the population may eventually facilitate a number of research and clinical issues, including prevention and control strategies.

It has been documented that early-onset female breast cancer differs from female breast cancer diagnosed at an older age in several ways. Firstly, female breast cancer diagnosed at a young age tends to have worse prognosis, namely fast-growing, high grade and hormone receptor-negative tumors (Anders et al. 2008; Fei et al. 2013; Partridge et al. 2010), and as a consequence a higher cause-specific mortality (Aebi et al. 2000; Albain et al. 1994; Anders et al. 2008; de la Rochefordiere et al. 1993; El Saghir et al. 2006; Holli and Isola 1997; Kollias et al. 1997).

Secondly, age-specific breast cancer incidence patterns differ between young and old women which usually show an inflection point around menopause (Adami et al. 1986; El Saghir et al. 2006; Nixon et al. 1994) where women have been reported to have a reduced risk of breast cancer (Fei et al. 2013).

Although previous studies have found the existence of two main breast cancer subpopulations according to age at onset, with the split point located around the menopause threshold (Anderson et al. 2002; Anderson and Matsuno 2006a; Anderson et al. 2006b; Tarone and Chu 2002; Yasui and Potter 1999), the etiology of these two groups is insufficiently understood and the question whether these subpopulations represent two different entities continues to be an open question; in addition, since it has been reported that changes in lifestyles over time have had influence in the occurrence of menarche, reproductive life and menopause in the US (Nichols et al. 2006), detecting and accounting for time-varying effects of age-at-diagnosis can provide insights on whether the characteristics at onset of the disease have also changed over time.

In this paper, the effects that temporal trend, clinicopathologic and sociodemographic variables have had on the distribution of age at diagnosis in a period of twenty years in the US is elucidated in order to understand the etiology of these two cancer subpopulations. A clustering method for identifying the two groups according to age at diagnosis in the presence of sociodemographic and clinicopathologic variables is implemented.

## 2 Materials and methods

### 2.1 Data source

**Figure 1:**
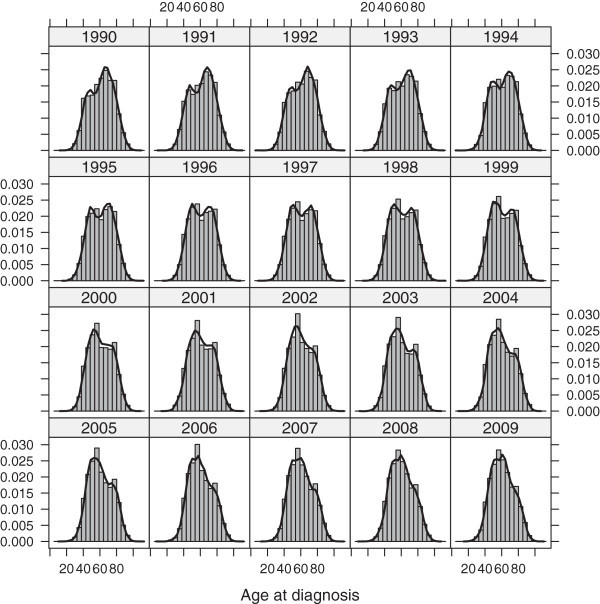
**Histogram and density curve of age-at-diagnosis for each year from 1990 to 2009.**

Surveillance, Epidemiology, and End Results (SEER) Program data of newly diagnosed cases of female breast cancer from the years 1990 to 2009 were analyzed. SEER is a high-quality, population-based incidence data covering up to 26% of the US population. During the period of study, *N* = 446,726 diagnosed cases of female breast cancer were registered in the nine SEER registries considered here, which include Atlanta, Connecticut, Detroit, Hawaii, Iowa, New Mexico, San Francisco-Oakland, Seattle-Puget Sound and Utah. Figure 1 depicts the histogram and kernel density curve of age-at-diagnosis for each year. Two key features are easily identified: a bimodal pattern and a notable change of both the shape and the location of the peaks. It can be noticed that, while the disease was more frequent in patients older than the inflection point during the first half of the 90’s, the diagnosis became more common amongst younger women in the following years.

The variables that were considered as predictors of age-at-diagnosis were trend, which was computed as the orthogonal polynomial base of first degree for year+(monthnumber−1)/12, and the factors that are defined in uppercase as follows: SITE, the histopathologic subtype: 1) duct carcinomas, obtained from codes 8500-8508 and 8521-8523 (80%), 2) lobular carcinomas, obtained from codes 8520 and 8524 (9%), and 3) Other (11%); ER, the estrogen receptor status: 1) positive (60%), and 2) negative or borderline (17%), with 23% missing; GRADE, tumor grade: 1) well differentiated (16%), 2) moderately differentiated (33%), and 3) poorly differentiated, undifferentiated or anaplastic (31%), with 20% missing; EXTENSION: 1) in situ or without underlying tumor or no evidence of it (18%), 2) confined to breast tissue and fat including nipple and/or areola (72%), and 3) Invasive components and further extension (8%), with 2% missing; LYMPH: 1) no lymph node involvement (70%), and 2) lymph node involvement (24%), with 6% missing; SIZE, tumor size: 1) less than 2cm (70%) and 2) 2cm or more (25%), with 5% missing; LATERALITY: 1) Right (49%), and 2) left (51%); RACE: 1) White (84%), 2) Black (9%) and 3) Asian or Pacific Islander (7%); and MARRIED, marital status at diagnosis: 1) Single (never married, 12%), and 2) Married (including common law), separated, divorced, widowed, unmarried or domestic partner (84%), with 4% missing. Only one-sided laterality cases were considered in the dataset since cases with breast cancer in both sides, which were rare (less than.5%), may have a different histopathology in each side. American Indians, Alaska natives and other unspecified races were not included in the study either (less than.1%). Time-dependent variables were created by forming interaction terms trend by factor.

### 2.2 Statistical methods

The random variable *Y* is defined as age at diagnosis and the model-based clustering technique employed here consists of a two-component mixture model to estimate both the underlying component distributions and the memberships of the two unlabelled groups. Specifically, the cumulative distribution function (c.d.f.) of the mixture model is defined by a weighted sum of two Gaussian component c.d.f’s as follows:
1

where *y* takes all real values, *Φ* represents the c.d.f. of the standard normal, and the unknown mixture proportion *π* (0≤*π*≤1) along with the two within-cluster means *μ*_1_ and *μ*_2_, and the two within-cluster variances  and  are to be estimated; here the clusters indexed as 1 and 2 will be referred to as the young cluster and the old cluster, respectively. In order to include auxiliary variables in the split-population model in equation 1, the two within-cluster means and variances are specified as functions of covariates as
2

where **x** is the vector of explanatory variables which includes the intercept, ***β***_*k*_ and ***γ***_*k*_ are vectors of coefficients and *k*=1,2. In a similar way, the mixture proportion is specified by a logit function as follows
3

where ***δ*** is the vector of coefficients. A similar formulation has been proposed in Villani et al. 2009. Finite mixture models have been extensively discussed in Everitt and Hand 1981 and McLachlan and Basford 1988.

The type of estimators used in this study are obtained with maximum likelihood; here, the log-likelihood of the data {*y*_1_,…,*y*_*n*_} is , where *g*(*y*;***θ***) is the density function corresponding to the c.d.f. of the mixture model in equation 1 and ***θ*** is the vector that contains all unknown parameters. The log-likelihood can be maximized using general purpose optimizers to find the maximum likelihood estimators and the standard errors. In this study, the function nlm of the R language was used to optimize *l*(***θ***). The likelihood surface in the analysis presented here was well behaved and the optimizing procedure always led to the same solution for different starting values. The assumptions made about the mixture model may be checked by calculating the conditional randomized quantile residuals proposed by Dunn and Smyth (1996), which are defined by , where  is the fitted cumulative distribution function and *i*=1,…,*n*. Since such residuals are exactly normal under the assumed model, some simple plots for checking that they are observed values of independent and standard normal random variates should indicate the quality of the fit.

The coding used to create the dummy variables corresponding to the levels of the factors was treatment contrasts (Chambers and Hastie 1992), which sets the coefficients of the baseline level in each categorical variable equal to 0; here, the baseline level is taken as the first category of the corresponding factor as described above. Since around 40% of the cases in the entire dataset contain at least a missing predictor, the principled method of dealing with the missing data employed here was multiple imputation. The inference procedure consisted of the generation of multiple stochastically “completed” datasets using the *mice* package in the R statistical language (van Buuren and Groothuis-Oudshoorn 2011), which uses a chained equations algorithm, then each completed data set was analyzed using the model for complete data, and finally the results were combined using Rubin’s rules (Rubin 1987).

Since the implementation of the chained equations algorithm is computationally expensive for large datasets, a random sample of size *n* = 20,000 was obtained, from which forty imputations were generated. The resulting five-number-summary of trend in the sample analyzed here was: minimum = −0.0132, 25th percentile = −0.0058, median=0.0003, 75th percentile=0.0061 and maximum=0.0116. It was assumed that the missing data mechanism is missing at random (MAR) (Little and Rubin 2002), which specifies that the probability that a data value is missing depends on values of variables that were actually measured. Including as many variables in the imputation model as possible yields multiple imputations that tend to minimize bias and make the MAR assumption more plausible, which reduces the need to make special adjustments for more complex missing data mechanisms (Schafer 1997).

The problem of variable selection in the mixture model was addressed with the “impute, then select” strategy, which involves initially performing multiple imputation and subsequently applying Bayesian variable selection to each of the enhanced datasets (Yang et al. 2005). The variables included in the final model appear in at least 50 per cent of the selected models obtained in the imputed datasets (Wood et al. 2008). To determine the most appropriate covariates to be included in the model of each imputed dataset, the Bayesian information criterion (BIC) (Schwarz 1978) was adopted as the main model choice criterion. If *n*_*p*_ denotes the number of parameters in the model and *n* the number of individuals in the dataset, the BIC criterion is to choose the model for which  is the smallest; here,  is the maximized log-likelihood function. Backward elimination was employed to arrive at the best fitting model in each imputed dataset. Both the variable selection process and the combined results were based on the forty enhanced datasets.

## 3 Results

When obtaining the most parsimonious model, it was found that, in the presence of trend and all factors, no time-varying variable had any impact on age-at-diagnosis, which suggests that the features at onset of the disease have not substantially changed during the twenty-year period studied here. Also, laterality was eliminated from the best fitting model, which indicates that it is not associated with the age at onset of breast cancer, confirming results from other studies (Weiss et al. 1996).

Although ER was selected in the most parsimonious model and it showed very significant effects when it was the only covariate included in the mixture model, it was noticed that, while its individual effects were non significant in the most parsimonious model, its presence made more significant most of the remaining variables. This suggests that the simultaneous inclusion of ER with other important auxiliary variables causes a problem of multicollinearity, in the sense that ER is correlated with some of the auxiliary variables. As a matter of fact, the association between ER and other important breast cancer histologic variables has been documented to be high, particularly with the grade of the tumor (Fisher et al. 1981; Lal et al. 2005). In this study, for samples of size 1000 of each enhanced dataset, the null hypothesis of independence between ER and GRADE was rejected for each sample at a significance level of 0.001 when performing the chi-squared contingency table test; in addition, the null hypothesis of ER being independent of SITE and SIZE was rejected for each sample at the significance level of 0.01. Since the presence of ER clearly overlaps the effects of other important variables, which causes overestimation and thereby an artificial inflation, it was decided that ER would not be included in the analysis.

Table 1 shows parameter estimates for the best fitting two-component Gaussian mixture model. The most significant coefficients correspond to trend, whose positive signs in the beta coefficients indicate that the two medians have moved to the right with time, which confirms that age at diagnosis has had a delaying effect with respect to time; in addition, the opposite signs of the gamma coefficients suggests that the young cluster has become more heterogeneous whilst the old cluster has become more homogeneous, and the negative delta coefficient implies that the age at onset has become more common for younger patients. When it comes to comparing the histopathologic subtypes, the coefficients show marked discrepancies between the duct and the lobular carcinomas. Broadly, lobular carcinomas tended to be diagnosed in women at earlier ages than women with duct carcinomas, particularly for the old cluster, and duct carcinomas have been more prevalent in older patients. There are no significant differences between duct carcinomas and other subtypes of carcinomas when categorized as a whole.

**Table 1:** **Parameter estimates (standard errors in brackets) of the best fitting two-component Gaussian mixture model for the regression of age-at-diagnosis of female breast cancer among US women diagnosed from 1990 to 2009 based on 40 imputations of a sample of size** ***n*** **= 20,000**

Covariate	***β***_1_	***β***_2_	***γ***_1_	***γ***_2_	***δ***
Intercept	50.29^∗∗∗^ (1.111)	71.91^∗∗∗^ (2.027)	4.039^∗∗∗^ (0.160)	4.172^∗∗∗^ (0.237)	−0.793^∗^ (0.337)
trend	347.3^∗∗∗^ (37.97)	363.1^∗∗∗^ (50.02)	25.92^∗∗∗^ (4.748)	−20.28^∗∗∗^ (6.118)	−73.57^∗∗∗^ (8.561)
SITE_2_	−1.901^∗∗^ (0.732)	−4.311^∗∗∗^ (1.149)	−0.387^∗∗^ (0.125)	0.359^∗∗^ (0.119)	0.713^∗∗^ (0.234)
SITE _3_	0.594 (0.820)	0.904 (0.812)	0.099 (0.100)	0.181 (0.100)	0.292 (0.156)
GRADE _2_	−0.007 (0.692)	0.351 (0.725)	0.211^∗^ (0.096)	−0.033 (0.094)	−0.257 (0.143)
GRADE _3_	−1.368 (0.744)	−0.172 (0.870)	0.287^∗∗^ (0.105)	0.009 (0.110)	−0.698^∗∗∗^ (0.160)
EXTENSION _2_	0.863 (0.890)	2.686 (1.693)	0.245^∗^ (0.121)	0.040 (0.199)	0.314 (0.293)
EXTENSION _3_	4.089^∗∗∗^ (1.189)	6.573^∗∗∗^ (1.781)	0.355^∗^ (0.159)	−0.147 (0.211)	0.455 (0.311)
LYMPH _2_	−2.380^∗∗∗^ (0.571)	−2.658^∗∗∗^ (0.704)	−0.100 (0.073)	0.113 (0.090)	−0.165 (0.122)
SIZE _2_	−0.540 (0.560)	2.060^∗∗^ (0.693)	0.125 (0.070)	0.088 (0.085)	−0.088 (0.120)
RACE _2_	−0.562 (0.738)	−1.174 (1.221)	0.057 (0.093)	−0.007 (0.147)	−0.433^∗^ (0.194)
RACE _3_	−0.708 (0.863)	−1.428 (1.151)	0.109 (0.108)	−0.133 (0.149)	−0.462^∗^ (0.211)
MARRIED _2_	1.961^∗∗∗^ (0.567)	−0.696 (0.979)	−0.185^∗^ (0.075)	0.005 (0.125)	0.845^∗∗∗^ (0.143)

^∗^ *p* - value < 0.05, ^∗∗^ *p* - value < 0.01, ^∗∗∗^ *p* - value < 0.001.

Coefficients corresponding to clinicopathologic variables indicate that high-grade cancers have been more frequent in the young cluster and invasive components and further extension tumors have been diagnosed at later ages than other levels of extension in both clusters, particularly for the old cluster. Also, contrary to other findings where no differences have been identified for lymph node status and tumor size amongst groups of age (Kollias et al. 1997), the results here indicate that, while lymph node involvement was more likely to be present in younger patients than those without it, bigger tumors were associated with older women in the old cluster, which was not the case amongst women in the young cluster.

In regard to sociodemographic characteristics, the coefficients suggest that Blacks and Asians or Pacific Islanders have tended to be more frequently diagnosed in the young cluster than their White counterparts; in addition, patients with breast cancer who were married at the time of diagnosis were more frequent in the older cluster, and women in the young cluster tended to be diagnosed at later ages than women with no partners, which may support the common belief that having a partner leads to a healthier lifestyle regarding substance use, physical activity and social support, delaying the onset of the disease amongst those susceptible (Bailey 2009).

A review of the randomized quantile residual plots for each enhanced dataset, which consisted of density, quantile-quantile, autoregressive and partial autoregressive plots, provided assurance that there is fairly normal distribution and independence of the residuals, which indicates that the model assumptions were met.

## 4 Discussion

This study has characterized the distribution of age at diagnosis in the North American population and examined factors that are related to this distribution. The results are based on a large sample from a population-based data-base collected in the US between 1990 and 2009. The sample analyzed here combines histopathology, and clinicopathologic, sociodemographic and trend variables. The comprehensive statistical analysis of the data along with the corresponding residual analysis confirms that such distribution is a mixture of two different populations. A strength of the present analysis is the number of patients from which trend, clinicopathologic and sociodemographic data are available; however, it is limited by the standardization of the clinicopathologic variables across time, the usual concerns related to registry data. This analysis hopefully provides a clearer understanding into the complexity driving age at onset of female breast cancer.

The most remarkable finding of the analysis was that temporal trend is the most important variable in determining the shapes, modes and probability of membership of the two populations over the 20-year follow up, which may suggest that, changes in reproductive and lifestyle factors, such as childbearing, eating habits (Tretli and Gaard 1996) and exposure to both exogenous and endogenous estrogens (Chen 2008), along with more frequent and different types of screening have had an important impact on the age at diagnosis. The main characteristic of trend was a steadily increasing age at diagnosis and a decreasing proportion of older women being diagnosed. Since no interaction trend by factor showed significant effects, it can be inferred that both clinicopathologic and sociodemographic variables have not been influenced enough by the embedded features that such trend may carry on their joint association with age at diagnosis. The results support findings from other research that duct and lobular carcinomas, the most common breast carcinomas, represent two different etiologies. As a matter of fact, other studies suggest that such carcinomas may be biologically different malignancies and have different risk factors (Pike et al. 1993).

There is much interest in estimating the inflection point between the two component densities in the mixture model in order to define a split-point that allows for the determination of the memberships in the two cancer populations in age-specific breast cancer incidence. In other studies, such split-point has been claimed to be around menopause (e.g. Anderson et al. 2006b; Yasui and Potter 1999). In this study, the p.d.f. of the mixture model has a complex interplay between the component means and variances and the mixture proportions in which the resulting p.d.f. is not necessarily bimodal, which makes difficult to analytically estimate the split-point for the different factor combinations and trend. Considering that menopause has also been delayed in North America due to the aforementioned lifestyle factors (Nichols et al. 2006) and that recent research supports a low breast cancer risk around this part of a woman’s life (Fei et al. 2013; Huang et al. 2011), it seems reasonable to speculate that the split-point is menopause. Nevertheless, the results obtained here indicate that the coefficients of sociodemographic and clinicopathologic variables in ***δ*** are important predictors for determining 1−*π*, the probability of membership in the young cluster, and *π*, the probability of membership in the old cluster. Such probabilities can be easily computed using the logit function in equation 3 and then be employed as a criterion in order to decide to which cluster a patient may belong, which could potentially improve the decision-making in patient management and survival.

The problem of multicollinearity in the model helped to confirm that ER status has a high degree of association with tumor grade, the histopathologic subtype and tumor size. Despite the recognised role of ER as a proxy for prognosis in medical decision making, it has been documented that hormone receptors such as ER are relatively weak predictors for determining outcome in patients diagnosed with breast cancer and are of limited clinical value in lymph node-negative cases (Mirza et al. 2002), which confirms that treatment guidelines based on ER alone are ill-defined. The results obtained here suggest that age at diagnosis, histopathologic subtype, tumor grade, tumor extension, lymph node involvement, tumor size, race and marital status at diagnosis along with other well established prognostic factors should jointly be used in order to build a conceptual framework for a more rational therapeutic approach to breast cancer. Given these considerations, the need for further research in this area is obvious.
